# High-Resolution Mapping Reveals Links of HP1 with Active and Inactive Chromatin Components

**DOI:** 10.1371/journal.pgen.0030038

**Published:** 2007-03-02

**Authors:** Elzo de Wit, Frauke Greil, Bas van Steensel

**Affiliations:** Department of Molecular Biology, Netherlands Cancer Institute, Amsterdam, The Netherlands; The Babraham Institute, United Kingdom

## Abstract

Heterochromatin protein 1 (HP1) is commonly seen as a key factor of repressive heterochromatin, even though a few genes are known to require HP1-chromatin for their expression. To obtain insight into the targeting of HP1 and its interplay with other chromatin components, we have mapped HP1-binding sites on Chromosomes 2 and 4 in *Drosophila* Kc cells using high-density oligonucleotide arrays and the DNA adenine methyltransferase identification (DamID) technique. The resulting high-resolution maps show that HP1 forms large domains in pericentric regions, but is targeted to single genes on chromosome arms. Intriguingly, HP1 shows a striking preference for exon-dense genes on chromosome arms. Furthermore, HP1 binds along entire transcription units, except for 5′ regions. Comparison with expression data shows that most of these genes are actively transcribed. HP1 target genes are also marked by the histone variant H3.3 and dimethylated histone 3 lysine 4 (H3K4me2), which are both typical of active chromatin. Interestingly, H3.3 deposition, which is usually observed along entire transcription units, is limited to the 5′ ends of HP1-bound genes. Thus, H3.3 and HP1 are mutually exclusive marks on active chromatin. Additionally, we observed that HP1-chromatin and Polycomb-chromatin are nonoverlapping, but often closely juxtaposed, suggesting an interplay between both types of chromatin. These results demonstrate that HP1-chromatin is transcriptionally active and has extensive links with several other chromatin components.

## Author Summary

In each of our cells, a variety of proteins helps to organize the very long DNA fibers into a more compacted structure termed chromatin. Several different types of chromatin exist. Some types of chromatin package DNA rather loosely and thereby allow the genes to be active. Other types, often referred to as heterochromatin, are thought to package the DNA into a condensed structure that prevents the genes from being active. Thus, the different types of chromatin together determine the “gene expression programs” of cells. To understand how this works, it is necessary to identify the genes that are packaged by a particular type of chromatin and to reveal how various chromatin proteins work together to achieve this. Here we present highly detailed maps of the DNA sequences that are packaged by a heterochromatin protein named HP1. The results show that HP1 preferentially binds along the genes themselves and much less to intergenic regions. Contrary to what was previously thought, most genes packaged by HP1 are active. Finally, the data suggest that HP1 may compete with other types of chromatin proteins. These results contribute to our fundamental understanding of the roles of chromatin packaging in gene regulation.

## Introduction

Originally identified as the densely staining regions of interphase nuclei [1], heterochromatin is now more precisely defined by its molecular components. Some of the best studied constituents of heterochromatin are Heterochromatin Protein 1 (HP1) and Su(var)3–9, which are both present in most eukaryotes. Su(var)3–9 is a histone methyltransferase that generates a histone 3 lysine 9 di- and tri-methylation (H3K9me2/3) mark [2]. This mark is recognized by HP1 and promotes retention of HP1 at chromatin [3,4]. HP1 interacts with a variety of proteins [5] and several of them, for instance Su(var)3–9 and Su(var)3–7, depend on HP1 for their heterochromatic targeting [6–9]. These interactions catalyze the formation of heterochromatin complexes.

Immunofluorescence microscopy of HP1 and Su(var)3–9 in *Drosophila* polytene chromosomes revealed that these proteins are abundant in pericentric regions and also are located at a few hundred discrete sites on the chromosome arms [10–12]. The limited resolution of light microscopy has precluded, in most cases, the identification of the sequences located in these bands. A much higher resolution can be obtained with the DNA adenine methyltransferase identification (DamID) technique, which was previously used in combination with cDNA array detection to systematically identify genes that are bound by HP1 and Su(var)3–9 [13,14].

HP1-containing heterochromatin is commonly known for its ability to repress gene expression. This notion is mostly based on studies with artificial reporter genes or with euchromatic genes that were integrated into heterochromatic regions by transgenesis or chromosomal rearrangements [15]. Paradoxically, some genes in *Drosophila* are naturally located in heterochromatin and require this heterochromatic environment for their correct expression [16–18]. Genome-wide mapping indicated that many more transcriptionally active genes in *Drosophila* may be bound by HP1 [14]. It is unclear how heterochromatin may repress certain genes while activating others [5,19].

How are heterochromatin complexes directed to specific parts of the genome? Experiments in fission yeast and Drosophila melanogaster suggest a role for the RNA interference (RNAi) machinery [20–22]. In this model small interfering RNAs direct HP1/Swi6 to its natural target sites. *Drosophila* mutants of RNAi machinery components show aberrant targeting of HP1 to euchromatic regions [23]. Thus, at least at some loci, the RNAi machinery is involved in heterochromatin deposition.

RNAi-dependent recruitment most likely accounts for the abundant heterochromatin deposition in highly repetitive regions of the genome [24–26]. Heterochromatin proteins are also known to preferentially associate with transposable elements (TEs) [13], and genetic evidence has suggested that certain TE types, such as *1360 (hoppel)* in *Drosophila*, might be genuine HP1 recruitment signals [27,28]. However, it was shown that *1360* elements are only bound by HP1 when they are embedded in a repeat-rich environment [13]. Most likely, cooperative binding to several closely neighboring repeats is required to create a stable heterochromatin complex.

Various observations point to additional, RNAi-independent signals in the formation of heterochromatin. For example, HP1 can be targeted to telomeres by direct binding of HP1 to telomeric DNA [29]. In higher eukaryotes, certain transcription factors may recruit heterochromatin components such as HP1 to promoters [30–32]. In *Drosophila*, HP1 has been found to be specifically enriched along the entire X chromosome in adult males, but not in females [13]. Furthermore, immunofluorescence microscopy and chromatin immunoprecipitation studies revealed the association of HP1 with the transcription units of activated heat-shock genes and several other transcribed loci [33,34]. Previously, we employed a genome-wide mapping approach in *Drosophila* to identify the genes that are bound by HP1 and Su(var)3–9 in vivo [13,14]. We found that on average these genes are significantly longer than genes not bound by heterochromatin proteins [13]. These observations suggest that transcribed genes, in particular long genes, harbor a targeting signal for heterochromatin complexes.

There is substantial evidence that heterochromatin can spread along the chromatin fiber in *cis* [35–37]. This spreading may be propagated by the interaction of HP1 with Su(var)3–9, which may in turn create new HP1-binding sites by methylation of H3K9 on neighboring nucleosomes [38]. Heterochromatin may be confined to specific regions by barrier sequences that prevent this spreading. Examples of such barriers are the *gypsy* insulator in *Drosophila* [39] and tRNA genes in fission yeast [40,41]. Alternatively, heterochromatin may be confined by the antagonistic action of neighboring euchromatin. In this case, a heterochromatin–euchromatin boundary may not be a fixed sequence element, but rather a dynamic boundary that relocates depending on the relative activity of euchromatic and heterochromatic factors [42,43].

A prerequisite for the thorough understanding of heterochromatin targeting mechanisms and functions is a high-resolution map of the in vivo location of heterochromatin factors in the genome. Such a map could uncover general sequence and chromatin features that are involved in heterochromatin targeting. Here, we used the DamID technology together with high-density genomic oligonucleotide arrays to generate chromosomal maps of HP1 binding in *Drosophila* cells, with a resolution of approximately 1–2 kb. Extensive analysis of this highly detailed map yielded several new insights into the genomic signals that govern the targeting of heterochromatin and revealed an intricate interplay between HP1 and other chromatin components.

## Results

### High-Resolution Mapping of HP1 by DamID

DamID has proven to be a powerful tool for the mapping of in vivo binding sites of chromatin proteins [13,14,44–50]. To obtain a high-resolution HP1-binding map we designed a genomic oligonucleotide array with a 60-bp probe every 100 bp, spanning the entire left arm and the first ~11 Mb of the right arm of Chromosome 2, as well as the first 2 Mb of the X chromosome and the entire fourth chromosome. This array design has been used previously to map the distribution of other chromatin proteins [48,51]. The resolution of DamID is estimated to be roughly 1–2 kb [47,52]. We omitted data from probes with repetitive sequences (i.e., sequences that occur more than once in the annotated fly genome), because these probes cannot be assigned to a specific genomic position.

**Figure 1:**
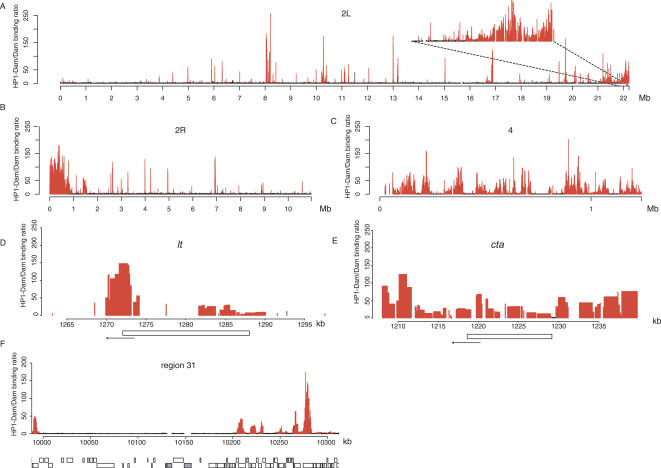
High-Resolution HP1-Binding Profiles (A and B) Maps of Chromosome 2, (C) Chromosome 4, (D) pericentric *light* gene, (E) pericentric *concertina* gene, and (F) cytological region 31. Inset in (A) shows a more detailed view of the centromere-proximal 0.4 Mb of 2L. Each stick represents the mean HP1–Dam/Dam binding ratio of a single GATC fragment, for one representative experiment. Fragments significantly bound by HP1 are marked in red, fragments not significantly bound by HP1 are shown in black. Gaps originate from nonunique sequences for which binding cannot reliably be determined. Positions of genes (open rectangles) and TEs (gray rectangles) are shown in D–F. Arrows in (D) and (E) indicate orientation of the genes.

We defined HP1 target loci by using an error model specifically designed for DamID on high-density oligonucleotide arrays (see Materials and Methods). This error model takes into account that Dam only methylates adenines in the sequence GATC, and that, therefore, DNA fragments demarcated by two GATC motifs represent the smallest units in the DamID mapping technique. Using this error model, 9,565 out of 94,202 fragments (~10%) are significantly bound by HP1. We generated maps to visualize the HP1-binding profile on Chromosomes 2 and 4 (Figure 1A–1C). These maps show abundant presence of HP1 in the pericentric regions of Chromosome 2 (Figure 1A and 1B) and on Chromosome 4 (Figure 1C), which is consistent with our earlier low-resolution maps [13,14] and immunofluorescence microscopy data [10,11]. More detailed inspection shows that HP1 on Chromosome 4 covers large domains that are approximately 10–100 kb in size, interrupted by regions not bound by HP1 (Figure 1C). The pericentric region of Chromosome 2 also shows variable levels of HP1 signal, but no discrete domains are visible as observed on Chromosome 4. Instead, binding occurs mostly in one large uninterrupted patch (Figure 1A, insert). In contrast, target sites along the chromosome arms are more focal and span usually only single genes (see below).

We selected several previously described heterochromatic regions, namely the two pericentric genes *light* and *concertina,* and the cytological region 31, for a more detailed view of HP1 binding. Both *light* and *concertina* reside in pericentric regions with elevated HP1 levels. Their transcription units and flanking intergenic regions are entirely covered by HP1, although the degree of binding varies (Figure 1D and 1E). We also examined cytological region 31, which shows strong enrichment for HP1 on polytene chromosomes [11]. Indeed, several genes in this region are bound by HP1. In contrast to what is observed for the pericentric genes, HP1 binding in region 31 is mostly restricted to the transcription units (Figure 1F). Earlier low-resolution DamID mapping [14] failed to detect enrichment of HP1 in region 31. This is now explained by the fact that most (6/8) genes in region 31 that are strongly bound by HP1 were not present on the cDNA array that was used in this previous study. This underscores the benefits of using high-density genomic tiling arrays. Altogether, in pericentric and nonpericentric regions we identified 357 genes with an average HP1 log_2_-ratio along the entire gene >1, out of 3,992 genes that are represented on our microarray. A total of 189 genes shows an average log_2_-ratio >2, and for several of the analyses described below we will use this more stringent cutoff.

### HP1 Binds to Active Genes

**Figure 2:**
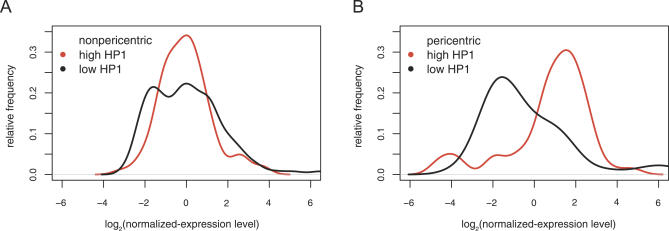
Most HP1-Bound Genes Are Actively Transcribed Density plot (smoothed histogram) showing the distribution of normalized expression levels of (A) nonpericentric and (B) pericentric genes that are strongly bound by HP1 (high HP1, average HP1 log_2_-ratio along entire gene >2 [red]) or genes with low or no binding by HP1 (low HP1, average HP1 log_2_-ratio along entire gene <2 [black]). Expression data were taken from Pickersgill et al. [50].

The binding of HP1 along transcription units suggests that HP1 may directly regulate the expression of these genes. To clarify the expression status of HP1 targets, we made use of transcription profiling data from *Drosophila* Kc cells obtained with 12 k cDNA arrays [50]. Because expression levels of HP1 targets differ depending on their chromosomal localization [14], we performed the analysis separately for genes on the chromosome arms and for pericentric genes (Figure 2A and 2B, respectively). For this purpose, pericentric genes were operationally defined as genes located on Chromosome 4 or less than 1 Mb from the centromere proximal ends of the sequenced parts of Chromosome 2. The remaining genes were classified as nonpericentric. In nonpericentric regions, the expression level distribution of genes that were strongly bound by HP1 (high-HP1 defined as an average HP1 log_2_-ratio along entire gene >2) is similar to that of genes that are weakly or not at all bound by HP1 (low-HP1 is defined as an average HP1 log_2_-ratio along entire gene <2). In contrast, pericentric genes strongly bound by HP1 are generally expressed at much higher levels than genes that are only weakly or not bound. Thus, HP1 target genes are active, but the expression levels indeed depend on the chromosomal localization: nonpericentric targets are expressed at average levels, whereas pericentric targets are expressed at high levels.

### HP1 Preferentially Binds to Exon-Dense Genes in Nonpericentric Regions

**Figure 3:**
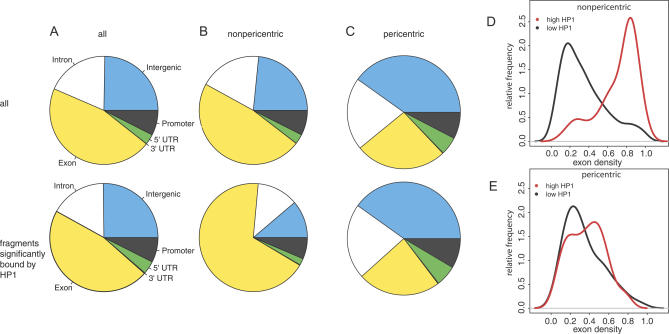
Preferential Binding of HP1 to Exon-Dense Genes (A–C) Pie charts showing the overlap of GATC fragments with promoters (black), 5′ UTRs (green), exons (yellow), introns (white), 3′ UTR (black), and intergenic regions (blue) for all fragments represented on the high-density oligonucleotide array (top) and only those fragments that are significantly bound by HP1 (bottom). This analysis was performed for (A) all fragments, (B) nonpericentric fragments, and (C) pericentric fragments. (D and E) Density plot showing the frequency distribution of exon densities (i.e., the fraction of sequence in transcription units that consists of exons) for genes with high HP1 levels (red) and genes with low HP1 levels (black), in (D) nonpericentric and (E) pericentric genes. Only genes with a length >5 kb were included in this analysis.

We wondered whether HP1 is preferentially located at transcribed regions or whether other genomic elements also have elevated HP1 levels. We therefore determined the frequency of HP1 binding to various genomic features (promoters, 5′ UTR, exons, introns, 3′ UTR, and intergenic regions). A first global analysis suggested that HP1 target sites are not enriched for specific genomic features (Figure 3A). However, when we repeated the analysis for pericentric and nonpericentric sites separately, we found that in nonpericentric regions HP1 has a strong preference for exons, while introns and intergenic regions are underrepresented in HP1-bound sequences (Figure 3B). No preference of HP1 for specific features was observed in pericentric regions (Figure 3C). These results point to at least partially distinct targeting mechanisms for HP1 in pericentric regions and on the chromosome arms.

The enrichment of HP1 in nonpericentric regions along transcribed genes and specifically at exons is surprising. This may be explained in two ways. First, HP1 could, within one and the same gene, bind specifically to exons, but not introns. Second, HP1 could be specifically targeted to genes that have a high density of exon sequence (and consequently a low density of introns). We therefore tested whether nonpericentric high-HP1 genes have a higher exon density than low-HP1 genes. Short genes, which are generally exon dense, were not included in this analysis. Analysis of genes with a size of >5 kb showed that nonpericentric high-HP1 genes indeed have a much higher exon density than nontargets (*p* = 2.4 × 10^−28^) (Figure 3D), while this is not the case for pericentric high-HP1 genes (*p* = 0.45) (Figure 3E). This suggests that in nonpericentric genes, a high density of exons may promote the recruitment of HP1. This preference for exon-dense genes is unlikely to be due to differences in base composition, because HP1 target genes and nontarget genes have nearly identical CG content (mean ± standard deviation is 0.467 ± 0.041 and 0.472 ± 0.049, respectively).

### Distribution of HP1 along Genes

**Figure 4:**
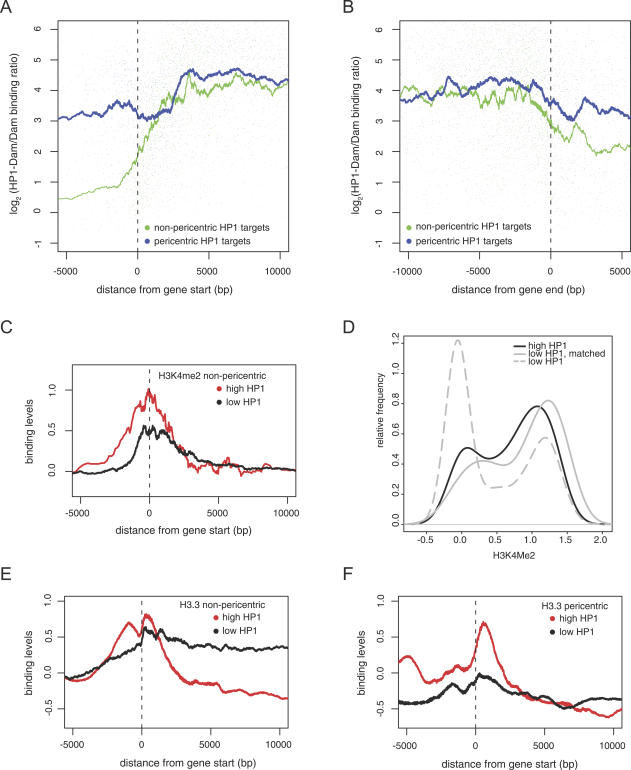
HP1 Binding Is Linked to H3K4me2 and Histone H3.3 Patterns Alignment of HP1-bound genes to (A) their TSSs and (B) the 3′ end of their transcription units. TSS-aligned genes include upstream regions up until the next upstream gene; 3′ end aligned genes include downstream regions until the next downstream gene. Curves show running mean (window size 100) of HP1-binding ratios (log_2_) for nonpericentric (green) and pericentric target genes (blue). (C) H3K4me2 levels of TSS-aligned genes in nonpericentric regions with high (red) or low (black) levels of HP1 as defined in Figure 2. H3K4me2 levels were taken from Schubeler et al. [53]. (D) Frequency distribution of H3K4me2 levels around the TSS (−500 to +1000 bp) for genes with high (black line) and low (gray lines) HP1 levels, either all genes (dotted gray line) or expression matched (solid gray line). (E and F) TSS alignment of H3.3 levels for genes with high (red) and low (black) HP1 levels in nonpericentric (E) and pericentric (F) regions. H3.3 data were taken from Mito et al. [51]. In (C), (E), and (F) running mean window sizes correspond to 2% of the total number of datapoints.

In order to elucidate the detailed distribution of HP1 in and around genes, we aligned all high-HP1 genes by their transcriptional start site (TSS) or by their 3′ ends and plotted a running mean of the HP1-binding log-ratios along the genes (Figure 4A and 4B). Again, we performed this analysis separately for pericentric and nonpericentric genes. This revealed that upstream of nonpericentric genes very little HP1 is bound. Within the genes, binding is low at the TSS and increases gradually until 1–2 kb into the gene, after which average HP1 levels reach a plateau that extends for the remainder of the gene (Figure 4A). Downstream of the 3′ ends the HP1 levels gradually decline (Figure 4B). Thus, in nonpericentric regions, HP1 is primarily associated with transcription units except for the first 1–2 kb. This pattern is qualitatively independent of the average binding level of HP1 along the entire gene (Figure S1A and S1B). Pericentric genes show a similar distribution pattern, but have much higher baseline levels of HP1 outside the transcription units. We also observed this pattern when we specifically analyzed genes on Chromosome 4 (data not shown). Taken together, HP1 shows characteristic binding patterns along genes that differ between pericentric and nonpericentric regions.

### Links between HP1 and Marks of Active Chromatin

As demonstrated above, HP1 is primarily associated with active genes. Genome-wide studies have recently shown that various other chromatin marks, such as specific histone modifications [53] and the histone variant H3.3 [51,54], are also enriched at active genes. To explore the relationships between HP1 and these marks, we re-analyzed available high-resolution chromosomal maps of dimethylated histone 3 lysine 4 (H3K4me2) and H3.3, which were generated in the *Drosophila* Kc cell line or in the closely related S2 cell line [51,53] (see Materials and Methods). H3K4me2 was previously found to be enriched at the 5′ regions of actively transcribed genes [54,55], whereas in *Drosophila* cells H3.3 was reported to be deposited during transcription along the entire transcribed region [51,54]. We compared the distribution of these two marks along high-HP1 genes and low-HP1 genes.

For the analysis of H3K4me2 distribution we focused on nonpericentric genes, because insufficient H3K4me2 data was available for pericentric genes. As expected, the average H3K4me2 pattern in 5′-aligned genes shows a clear peak centered around the TSS (Figure 4C). Strikingly, this peak is much more pronounced in high-HP1 genes than in low-HP1 genes.

Quantitative analysis of the H3K4me2 levels between −500 and +1000 bp (Figure 4D) revealed a clear bimodal distribution of this histone mark. The vast majority of genes with high HP1 binding have high H3K4me2 levels, while genes with low HP1 binding display more frequently low H3K4me2 levels around the TSS. We considered the possibility that H3K4me2 is present only at genes with expression levels above a certain threshold. The correlation between H3K4me2 status and HP1 binding could then be explained by the fact that virtually all HP1-bound genes are “on,” while a considerable fraction of the genes that lack HP1 are completely “off.” (Note in Figure 2A that low-HP1 genes have a broader distribution of expression levels than high-HP1 genes). To test this, we repeated the analysis of H3K4me2 levels for a subset of low-HP1 genes that were selected to have the same distribution of expression levels as high-HP1 genes (see Materials and Methods). Indeed, the H3K4me2 levels of this expression-matched set (Figure 4D, solid gray line) showed no significant difference to those of HP1-bound genes. Thus, the high average levels of H3K4me2 in HP1-associated genes are most likely explained by the fact that these genes are almost never completely “off” and may not be related to the presence of HP1 per se.

Next, we investigated whether there is a link between H3.3 deposition and HP1 levels (Figure 4E and 4F). We observed an overall enrichment of H3.3 near the TSS, which is consistent with previous observations [51,54]. However, we found pronounced differences in the H3.3 distribution depending on the level of HP1 binding and in conjunction with the chromosomal location of genes. Nonpericentric genes with low levels of HP1 show strong enrichment of H3.3 along the entire transcription unit, and also a moderate enrichment in a region about 0–3 kb upstream of the gene (Figure 4E). In contrast, at nonpericentric genes with high levels of HP1, H3.3 is confined to two clear peaks on either side of the TSS (Figure 4E); no enrichment of H3.3 is seen further towards the 3′ parts of these transcription units, suggesting that H3.3 deposition along transcribed regions is incompatible with HP1 binding. Indeed, the amount of H3.3 along the transcription units is inversely proportional to the HP1 levels (Figure S1C). In pericentric genes, the distribution of H3.3 is strikingly different (Figure 4F and Figure S1D): high-HP1 genes show only a single peak of H3.3 just downstream of the TSS, while low-HP1 genes are almost devoid of H3.3 along their entire length. Taken together, these results reveal an intricate relationship between HP1 and H3.3 that depends on chromosomal location (see Discussion).

### Juxtaposition of HP1 and Polycomb Domains

Polycomb (Pc) is part of a type of repressive chromatin that is sometimes also referred to as heterochromatin. Most evidence indicates that HP1-marked heterochromatin and Pc-containing chromatin are distinct and targeted to different genomic regions [48,56,57]. However, other data suggest cross-talk between components of the two types of chromatin and partial overlap of their target loci [43,58–60]. We therefore compared our high-resolution binding map of HP1 to that of *Drosophila* Pc, which was recently constructed by DamID in Kc cells using the same high-density arrays [48].

**Figure 5:**
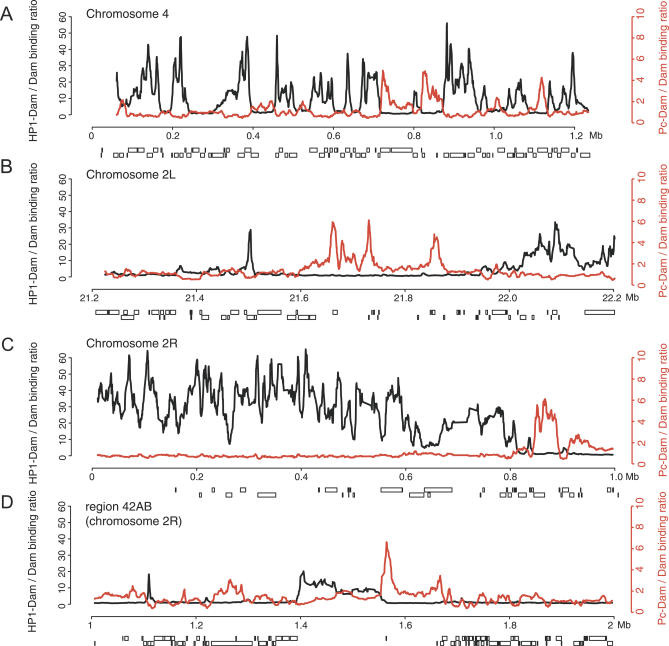
HP1 and Polycomb Form Two Distinct, Nonoverlapping Chromatin Domains That Are Often in Close Proximity to Each Other Running mean (window size 20 GATC fragments) of HP1-Dam/Dam–binding ratios (black) and Pc-Dam/Dam–binding ratios (red) of (A) Chromosome 4; (B and C) pericentric regions of Chromosome 2; and (D) a repeat-rich region on the right arm of Chromosome 2 (cytological region 42AB). Positions of genes are indicated below each graph.

We found that HP1 and Pc clearly bind to distinct regions (Figure 5A–5D, black and red lines, respectively). In fact, the Pc signal in HP1 domains is frequently lower than the general baseline of Pc levels in regions where neither of the two proteins is enriched (Figure 5A and 5B), suggesting that Pc may be actively excluded from HP1 domains.

Strikingly, in pericentric regions and on the fourth chromosome, HP1 and Pc domains are frequently in close proximity to each other, with rather sharp transitions, giving the impression of a strict demarcation between these domains (Figure 5A–5C and Figure S2). We did not observe this close juxtaposition of HP1 and Pc domains along the chromosome arms, with the exception of a repeat-rich region on the right arm of Chromosome 2 (Figure 5D and Figure S2). The frequent propinquity of HP1 and Pc domains in pericentric regions and on the fourth chromosome suggests that both domains may interact (see Discussion).

### HP1 Binding to Individual Copies of TEs

**Figure 6:**
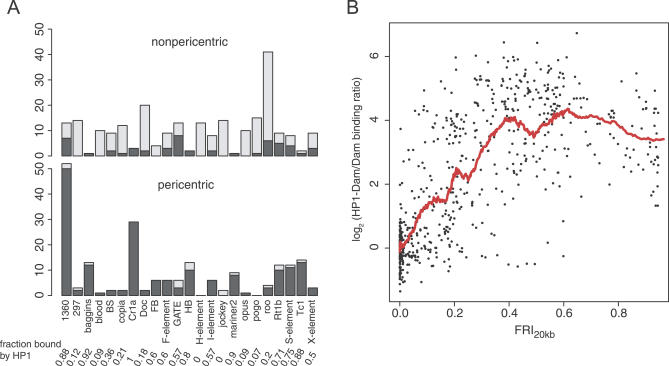
HP1 Binding to Individual Transposon Copies (A) Frequencies of copies of the different TE types that are target of HP1 (dark gray) in nonpericentric (top) and repeat-rich pericentric (bottom) regions. A TE copy was counted as an HP1 target if, in the unique flanking 1 kb on each side of the TE, at least one GATC fragment was significantly bound by HP1. (B) HP1-Dam/Dam–binding ratios at unique sequences within 1 kb of a TE are plotted as a function of the FRI_20kb_ (see main text). Running mean with window size 20 is shown for HP1 binding as a function of the FRI_20kb_ (red line).

Intergenic sequences in pericentric regions show extensive binding of HP1. Because these regions are rich in TEs, and because many TEs bind HP1 [13,14], we reasoned that TEs may provide important nucleation sites for pericentric heterochromatin. Microarray studies of repetitive sequences such as TEs are complicated by the fact that probes with homology to repeats cannot discern between individual repeat copies and thus only provide population averages. To obtain an estimate of HP1 binding to individual TE copies, we took advantage of the fact that methylation by tethered Dam spreads in *cis* over about 1–2 kb [47,52] and analyzed the level of targeted methylation detected at unique sequences within 1 kb from each TE integration site. These data indicate that the majority of TE copies in pericentric regions are bound by HP1. Strikingly, nearly all TE copies that have an FRI_20kb_ higher than 0.4 have elevated HP1 (log_2_-ratios >1, Figure 6B). While this is much less frequently the case for TE copies in nonpericentric regions (Figure 6A).

A previous statistical analysis based on low-resolution DamID data [13] predicted that binding of HP1 to an individual TE requires that this element is located in a repeat-dense environment. Measurements of two individual *1360* elements supported this prediction. To validate this on a larger scale we compared the estimated HP1 binding at all probed individual TE insertions to the local repeat density. We used the previously described flanking repeat index (FRI_20kb_, i.e., the fraction of repeat sequence within 20 kb of DNA at each side of the TE) as a measure of the local repeat density [13]. This demonstrates that HP1 has a strong preference for TEs that are located in repeat-dense regions, supporting a model in which multiple neighboring repetitive sequences recruit HP1 in a cooperative manner [13]. Strikingly, nearly all TE copies that have an FRI_20kb_ higher than 0.4 have elevated HP1 levels (log_2_-ratios > 1, Figure 6B).

## Discussion

By mapping of HP1 binding at high resolution we show that genes that naturally reside in HP1-chromatin are transcriptionally active. Additionally, we obtained new insights into the targeting of HP1 and the interplay of HP1 with other chromatin components.

### HP1 Binding and Gene Activity

HP1-containing chromatin is often assumed to be compacted and repressive. This notion is primarily based on classical studies with euchromatic reporter genes that were relocated to a heterochromatic region. Such reporter genes often show position effect variegation, and the many examples of this effect have contributed to the perception that gene repression is a general feature of heterochromatin. Interestingly, some studies several years ago demonstrated that certain genes naturally embedded in heterochromatin are transcriptionally active and even require a heterochromatic environment for their expression [18,61,62] (reviewed in [19]). Immunofluorescent labeling of polytene chromosomes also indicated that some active genes on the chromosome arms recruit HP1 [33]. Our mapping data show that the association of HP1 with active genes is the general rule rather than an exception. We find that HP1-bound genes are on average equally active as non-bound genes on the chromosome arms, while in pericentric regions the HP1 target genes are even highly transcribed.

In a previous study, we also reported the high activity of pericentric HP1 target genes [14]; modestly lower expression levels were found for nonpericentric HP1-bound genes. Here we cannot confirm the somewhat reduced expression of nonpericentric HP1-bound genes. We believe that our current genomic tiling array gives a more reliable view than the previously used cDNA array [14], which may have contained a slightly biased selection of genes. In addition, the current use of a DNA reference to normalize the expression microarray signals [50], which was not done in our earlier study [14], is likely to provide a more accurate estimate of expression levels. The strong enrichment for H3K4me2, a marker of active TSSs, confirms that most HP1 target genes are indeed transcribed.

Several other recent reports support the view that heterochromatin marked by HP1 and H3K9 methylation is not transcriptionally silent. Mammalian HP1 homologs together with di- and tri-methylated H3K9 have also been found at active transcription units [34,63–65], and in fission yeast the deposition of the HP1 homolog Swi6 is linked to the transcription machinery [66,67]. Thus, the association of HP1 and H3K9me2/3 with active genes appears to be a general phenomenon. Future elucidation of the molecular structure of HP1-containing chromatin may provide an explanation for the position effect variegation paradox, i.e., why natural target genes of HP1 are expressed, while euchromatic reporter genes tend to become repressed upon integration into heterochromatic regions.

### Detailed HP1-Binding Patterns

Comparison of the HP1 patterns in pericentric and nonpericentric regions revealed differences in HP1 targeting between these different chromosomal locations. In nonpericentric regions HP1 binding is mostly restricted to transcription units, implying a targeting signal inside these transcription units. Possibly, HP1 is recruited cotranscriptionally, in agreement with observations that RNA Polymerase II is required for RNAi-dependent heterochromatin assembly in fission yeast [66,67]. In pericentric regions, HP1 associates with transcription units as well as intergenic regions. This additional intergenic targeting of HP1 in pericentric regions is likely due to the high density of repeats.

HP1 targeting to nonpericentric transcribed regions is linked to a high exon density. The mechanisms and reasons for this surprising exon bias are unclear. We previously reported a preference of HP1 for long genes [13], which we confirmed by analysis of the high-resolution data presented here (unpublished data). Thus, HP1 appears to associate with genes that share specific structural features. We speculate that HP1 is involved in transcriptional elongation, alternative splicing, or other aspects of RNA metabolism, similar to what has recently been observed for the human chromatin remodeling protein Brm [68].

The genomic distribution of HP1 in *Drosophila* shows striking similarities with that of DNA methylation in *Arabidopsis* [69]. In this plant, methylation is found predominantly on transcription units, with a very similar distribution along genes as seen for HP1 in *Drosophila*. Moreover, methylation occurs predominantly in genes with moderate expression levels, as is the case for HP1. It was proposed that DNA methylation serves to suppress the activity of cryptic promoters in transcribed regions [69]. HP1 may be involved in a similar function.

Previous studies in various organisms have found HP1 to be bound to certain promoters [30–32,70,71]. Our data indicate that, at least in *Drosophila*, HP1 is predominantly associated with transcription units, but rarely with promoters. We cannot exclude that a small set of promoters is bound and regulated by HP1.

### Interplay of HP1 with Active Chromatin Components

The typical distribution of HP1 along a transcription unit shows low binding at the TSS and a gradual increase downstream of the TSS. A similar HP1-binding pattern along genes was recently observed in humans [65], suggesting that the targeting mechanism of HP1 proteins is at least partially conserved during evolution. The presence of H3K4me2 (a mark of active chromatin) at HP1 bound genes is in accordance with our observations that HP1 target genes are expressed.

Previous studies in *Drosophila* indicated that histone H3.3 is deposited along transcribed regions by a transcription-coupled mechanism [51,54,72]. Our re-analysis of available H3.3 maps confirms that this is the case for genes not bound by HP1, but shows that deposition of H3.3 is restricted to the first ~2 kb of transcription units of genes bound by HP1. HP1 binding is typically low in these same regions, indicating that H3.3 and HP1 are mutually exclusive marks of active genes. This apparent incompatibility is consistent with the observation that H3.3 in *Drosophila* cells is enriched in acetylation and depleted in dimethylation of lysine 9 [73] and therefore is predicted to have low affinity for HP1 [3,4]. Mammalian HP1 homologs together with dimethylated H3K9 also associate with many active transcription units [34,63–65], and H3.3 has been found preferentially at the 5′ ends of mouse genes [74], but it is not known whether these proteins are also mutually exclusive in mammalian genes. Interestingly, in nonpericentric regions we noted that HP1-bound genes are marked by an additional peak of H3.3 just upstream of the TSS. The function of the upstream deposition of H3.3 is unclear, but it underscores the interplay between this histone variant and HP1-containing chromatin. Future studies may reveal the causal relationships in this interplay.

The genomic binding pattern of HP1c, a homolog of HP1, was recently also mapped by DamID [14,49]. Interestingly, the distribution of HP1c is very different from that of HP1. HP1c is not enriched in pericentric regions, nor is it located along transcription units, but rather shows a more focal distribution. HP1c foci often correspond to discrete sites where many transcription factors and other regulatory proteins congregate (hotspots) [49]. These hotspots are preferentially located in or near highly active genes. Thus, like HP1, HP1c appears to be linked to transcription activity rather than repression, but it shows a different mode of binding, which strongly suggests a different molecular function.

### Relationship between HP1 and Pc Domains

We found that Pc and HP1 domains are often located directly next to each other, particularly in pericentric regions and on the fourth chromosome. This frequent juxtaposition of HP1 and Pc domains suggests that these two types of chromatin domains may interact. For example, HP1 domains may confine Pc domains by preventing the *cis*-spreading of Pc protein complexes; likewise (and perhaps simultaneously), Pc domains may set limits to HP1 domains. Competition between the two chromatin types, which appear to be mutually exclusive, could determine the position of the boundary between these domains. It is interesting to note that genes in Pc domains are typically repressed [48], while HP1-associated genes are mostly active. Thus, dynamic relocation of the boundary between these two types of domains could be a means to regulate genes that are located close to this boundary.

Alternatively, HP1 and Pc domains may be separated by static boundaries, encoded by specific sequence elements that recruit insulator proteins. Several proteins with insulator activity have been described [75–77]. DamID mapping of these proteins is likely to provide insight into their putative role in separating HP1-marked heterochromatin from other chromatin types such as Pc-domains.

## Materials and Methods

### DamID and array design.

DamID was performed as described [14] in embryonal D. melanogaster Kc_167_ cells grown in BPYE medium (Shields and Sang M3 insect medium supplemented with 2.5 g/l bactopeptone, 1g/l yeast extract, and 5% heat-inactivated fetal calf serum). HP1-Dam methylated fragments as well as Dam-only methylated fragments were amplified by PCR and subsequently hybridized to high-density oligonucleotide arrays containing a 60-bp probe every 100 bp. These arrays cover the entire Chromosome 2L, 10 Mb of Chromosome 2R, 2 Mb of the X chromosome, and Chromosome 4 [51]. Labeling of methylated DNA fragments, hybridization, and scanning of arrays was performed by NimbleGen (http://www.nimblegen.com). Probes that gave more than one significant alignment with the *Drosophila* genome sequence using MEGABLAST (standard settings) [78] were marked as repeats and omitted from subsequent analyses.

### Normalization procedures.

We performed global-intensity–dependent normalization using the robust scatterplot smoother “lowess” [79]. For this procedure, we omitted probes that did not have any sequence overlap with sequences in the genome or had multiple alignments in the genome.

### HP1 target definition.

During DamID profiling, the Dam enzyme of the HP1–Dam fusion and the Dam-only control proteins methylates adenosines in GATC sequences. Fragments between two methylated GATC sequences are subsequently amplified, and HP1-Dam/Dam ratios are calculated to determine the HP1-binding sites [14]. Thus, the smallest unit of a DamID profile is the region between two GATC sequences. Therefore, HP1-binding log_2_-ratios of all 60-bp probes originating from the same GATC restriction fragment were averaged to obtain a single HP1-binding log_2_-ratio for this GATC fragment. If a probe overlapped with a restriction site, the probe was assigned to the GATC fragment with the longest sequence overlap. The averaged log_2_-ratios were used for all subsequent analyses.

To determine GATC fragments that were significant targets of HP1, the following DamID-specific error model was developed: first we estimated the variance of the DamID log_2_-ratios based on all negative values, similarly to the ChIPOTle algorithm [80]. We used this approach instead of simply calculating standard deviations, because DamID profiles have an intrinsic bias towards positive values, skewing the standard deviations. Second, every HP1-binding log_2_-ratio was divided by this estimated variance. This yielded a Z-score, from which a nominal *p*-value could be calculated. Third, the *p*-values of three independent experiments were combined [81], and finally, these combined *p*-values were corrected for multiple testing using the Benjamini-Hochberg method [82]. GATC fragments with *p* < 0.01 were classified as HP1 targets.

### Definition of pericentric and nonpericentric regions.

Pericentric regions are defined as the centromere-proximal 1Mb of sequence on the left and right arms of Chromosome 2, as well as all of Chromosome 4. Sequences were taken from release 3 of the *Drosophila* genome (BDGP).

### Definition of intragenic and intergenic components.

Each GATC fragment was assigned to one of the following genomic features: promoters, 5′ UTRs, exons, introns, 3′ UTR, and intergenic regions. Positions of these features were obtained from the BDGP annotation, release 3.2.2. Promoters were defined as 2 kb of intergenic sequence immediately upstream of each TSS. If a GATC fragment overlapped with multiple genomic features, the following hierarchy in assigning fragment identity was applied: exons had the highest precedence (i.e., if a GATC fragment overlapped with exon sequence it was assigned to the exon bin), 5′ and 3′ UTR sequences had precedence over promoters and intergenic sequences, and finally, promoters had precedence over other intergenic sequences. Intergenic sequences and introns thus had the lowest precedence. Exon density of genes is defined as the fraction of a gene that is exon sequence.

### Alignments of HP1 target genes.

Start and end position of all tested genes (transcription units) were defined according to BDGP annotation, release 3.2.2. Assignment of GATC fragments to genes and alignment to the gene end or start was performed using custom perl scripts (available on request). For TSS alignments we included upstream regions up until the preceding upstream gene; conversely, for 3′ end alignments we included downstream regions until the following downstream gene.

### Expression matching of HP1 target genes and nontarget genes.

A set of nontarget genes that have similar expression levels as HP1 target genes was selected as follows. All genes were ranked based on their expression level. Next, genes that were ranked directly above and below an HP1 target gene were chosen as expression-matched nontarget genes. If the gene ranked above or below was also an HP1 target gene, the two genes ranked above and below these two HP1 target genes were chosen as expression matched nontarget genes.

### Comparison of HP1 and other chromatin proteins.

H3K4me2 data [53] was obtained from the S. Bell laboratory Web site (http://chromosome2l.mit.edu). Polycomb protein data are from Tolhuis et al. [48]. Histone H3.3 data are from Mito et al. [51]. H3.3 maps were generated in S2 cells, which like Kc cells are of embryonic origin. Analysis of a published expression profiling study [83] indicates that ~94% of HP1 target genes (106 out of 113 present in the expression profile) show less than 2-fold difference in expression between Kc and S2 cells. This is roughly the same for nontarget genes (92%). We therefore feel that the comparison of the data from the two cell types is justified.

### Definition of FRI.

The FRI_20kb_ was defined as previously described [13]. Briefly, we calculated the fraction of nonunique sequence in a region 20 kb downstream of a TE and 20 kb upstream of a TE, including the TE itself.

### URLs.

All statistical analyses were performed using the R software environment (http://www.r-project.org). *Drosophila* genome annotation release 3.2.2 was downloaded from http://flybase.net.

## Supporting Information

Figure S1 Aligned Binding Profiles of HP1 and H3.3 at Various Cut-Off Levels of HP1 (2.9 MB AI).

Figure S2 Detailed Maps of HP1-Polycomb Transitions on Chromosome 4 and 2R (307 KB PDF)

### Accession Numbers

The Gene Expression Omnibus (http://www.ncbi.nlm.nih.gov/geo) accession number for the HP1 binding data discussed is GSE6564.

## Abbreviations

Dam: DNA adenine methyltransferase
DamID: DNA adenine methyltransferase identification
FRI: flanking repeat index
H3K4me2: dimethylated histone 3 lysine 4
HP1: Heterochromatin Protein 1
Pc: Polycomb
RNAi: RNA interference
TE: transposable element
TSS: transcriptional start site
