# Supplementary material for: High-Resolution Mapping Reveals Links of HP1 with Active and Inactive Chromatin Components
Source: PLoS Genet. 2007 Mar 2;3(3):e38. doi: 10.1371/journal.pgen.0030038 (PMC1808074; doi:10.1371/journal.pgen.0030038)
Supplement: Supplementary file 1 [file pgen.0030038.sg001.pdf]

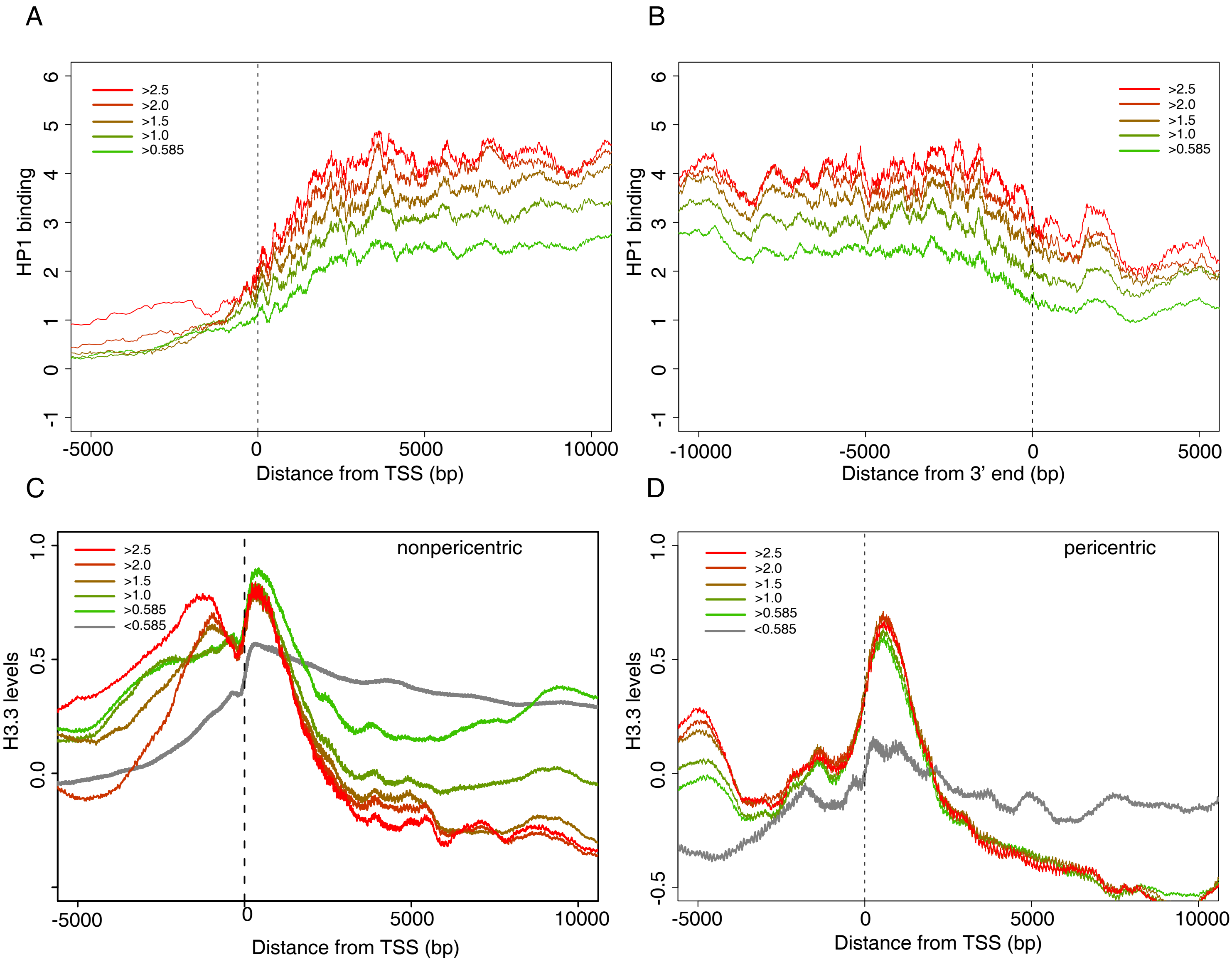

Figure S1: Aligned binding profiles of HP1 and H3.3 at various cut-off levels of HP1. Alignments were made of the genes that have average log_2 HP1 levels above the indicated cut-off. The lines represent sliding window means of the aligned data, with the window size being $1/50^{th}$ of the total number of datapoints for each category.
(A-B) HP1 levels for nonpericentric genes, aligned to (A) the transcription start sites or (B) the 3' ends.
(C-D) TSS-aligned H3.3 levels at various cut-offs for HP1, for (C) non-pericentric and (D) pericentric genes. The gray lines represent the averaged profile of all genes below the lowest cut-off.
