# Supplementary material for: High-Resolution Mapping Reveals Links of HP1 with Active and Inactive Chromatin Components
Source: PLoS Genet. 2007 Mar 2;3(3):e38. doi: 10.1371/journal.pgen.0030038 (PMC1808074; doi:10.1371/journal.pgen.0030038)
Supplement: Supplementary file 2 [file pgen.0030038.sg002.pdf]

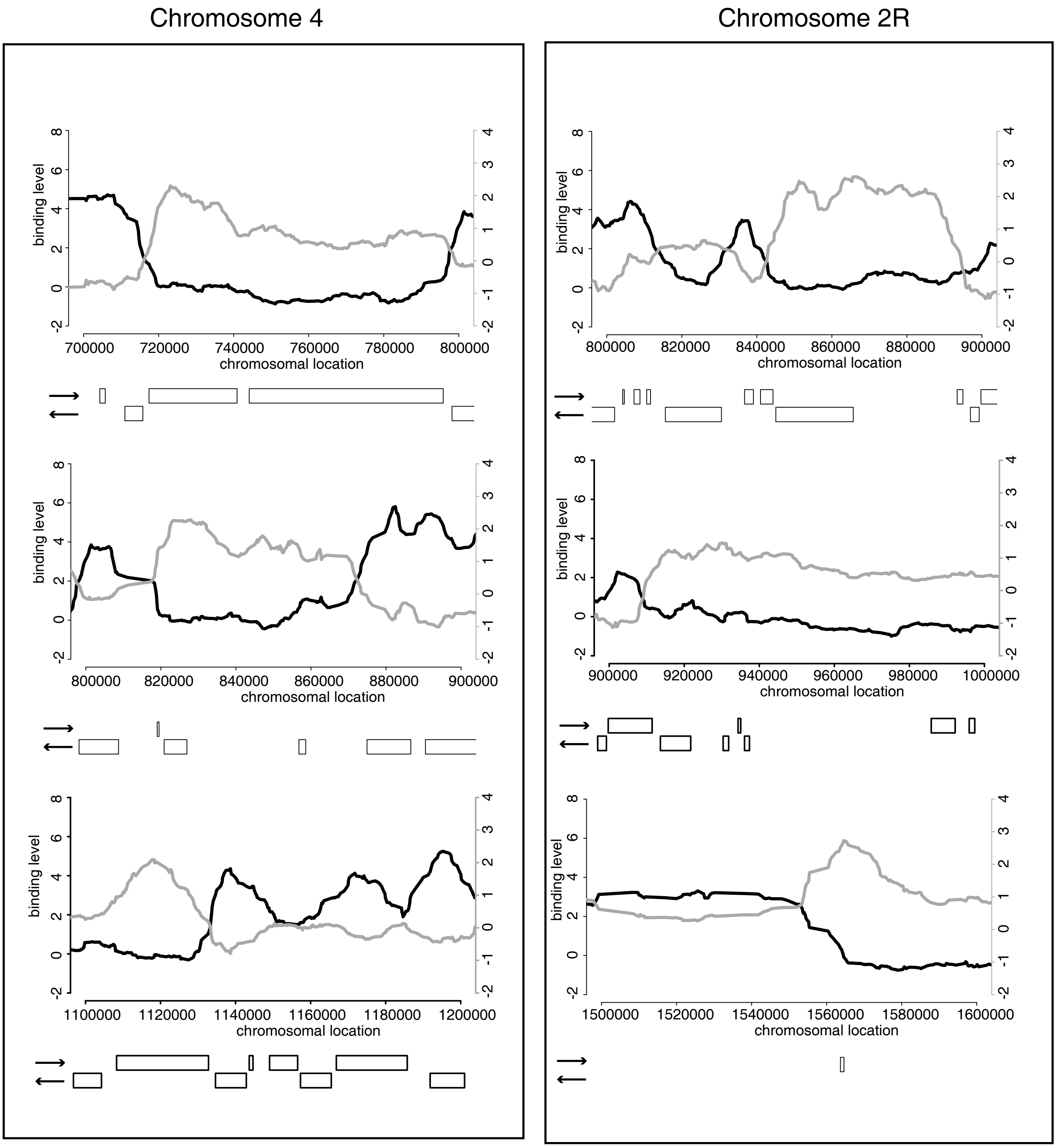

Figure S2: Detailed maps of HP1-Polycomb transitions on chromosome 4 and 2R. The black line represents the running mean of HP1 binding with a window size of 20. The gray line depicts a running mean of Polycomb binding with the same window size. Below every detailed map the genes are shown, arrows indicate the direction of transcription.
